# It Works, But For Whom? Examining Racial Bias in Carding Experiences and Acceptance of a County Identification Card

**DOI:** 10.1089/heq.2018.0022

**Published:** 2018-09-25

**Authors:** Alana M.W. LeBrón, Keta Cowan, William D. Lopez, Nicole L. Novak, Maria Ibarra-Frayre, Jorge Delva

**Affiliations:** ^1^ Department of Population Health and Disease Prevention, University of California, Irvine, Irvine, California.; ^2^ Department of Chicano/Latino Studies, University of California, Irvine, Irvine, California.; ^3^ Synod Community Services, Ypsilanti, Michigan.; ^4^ School of Public Health University of Michigan and National Center for Institutional Diversity, Ann Arbor, Michigan.; ^5^ Prevention Research Center, University of Iowa, Iowa City, Iowa.; ^6^ School of Social Work, Boston University, Boston, Massachusetts.

**Keywords:** driver's license, government-issued ID, health inequities, local government-issued ID, REAL ID Act, social determinants of health

## Abstract

**Purpose:** Policies that restrict access to U.S. government-issued photo identification (ID) cards adversely affect multiple marginalized communities. This context impedes access to health-promoting resources that increasingly require government-issued IDs and exacerbates health inequities. In 2015, Washtenaw County, Michigan, implemented the Washtenaw ID to improve access to resources contingent upon having an ID. We employed an audit study to examine whether Washtenaw ID users experienced racially biased treatment in carding experiences and acceptance of the Washtenaw ID.

**Methods:** Seven 25- to 32-year-old mystery shoppers (two Latina, three black, and two white women) attempted to purchase a standardized basket of goods, including an age-restricted item in Washtenaw County stores (*n*=130 shopping experiences). We examined whether experiences of being asked for ID and acceptance of the Washtenaw ID varied by race/ethnicity.

**Results:** Each shopper visited 9–22 stores. Shoppers were asked for ID in 63.1% of shopping experiences. Of these, the Washtenaw ID was accepted 91.5% of the time. Among those who were asked for ID, a higher percentage of Latina (16.0%) shoppers had their Washtenaw IDs rejected than black (6.3%) and white (4.0%) shoppers, although differences were not statistically significant (*p*=0.27). Latina shoppers had 2.9 times the odds of receiving a comment about their Washtenaw ID relative to white shoppers (OR=2.92, *p*=0.08), comments that were nonpositive.

**Conclusion:** Local IDs may improve access to resources contingent upon having an ID. However, racialization processes, including anti-immigrant sentiments, may inhibit the mitigating goal of local IDs. Continued attention to the health equity impacts of equity-driven interventions is warranted.

## Introduction

Twenty-first century federal policies, and bureaucratic institutions' interpretations of such policies, have increased the need for a U.S. government-issued photo identification (ID) card (henceforth, *government-issued ID*) to access social, economic, and healthcare resources that promote health and health equity.^1^ For example, the 2001 PATRIOT Act requires verification of customers' identities for financial transactions.^2^ Consequently, many financial institutions have implemented policies requiring a current government-issued ID. Other institutions have adopted similar de jure or de facto government-issued ID requirements that affect the social determinants of health.

Health-promoting resources with ID requirements may include housing (e.g., applying for leases and viewing housing); healthcare (e.g., proving health insurance status); pharmaceutical and financial (e.g., opening bank accounts and cashing checks)^3^; political enfranchisement (e.g., voting)^4^; governmental records (e.g., getting birth certificates); goods and services,^5^ age-restricted goods, and community resources (e.g., library cards and food banks)^3,6^; and governmental safety net programs (e.g., Medicaid and food stamps).^1,7^ IDs are also needed to safely prove identity to law enforcement to prevent detention or interactions with other law enforcement agencies.^1,3^ Because IDs now serve as gateways to health-promoting resources, ID policy is health policy.

Restrictive ID policies render access to health-promoting resources a privilege only available to those with a current government-issued ID. For example, the 2005 REAL ID Act requires proof of authorized U.S. presence in order for state-issued driver's licenses and state IDs to be used for federal identification purposes.^8^ State responses to this federal policy have tightened criteria for obtaining IDs. In response, 40 U.S. states, including Michigan, currently deny driver's licenses and state IDs to persons who cannot prove their authorized U.S. presence.^9^

Notably, this context enhances inequities between individuals who have government-issued ID and those who do not. Multiple marginalized communities, including racial/ethnic minority, immigrant, low-income, formerly incarcerated, transgender, those with chronic mental illness, those experiencing a catastrophic event or housing instability, and elderly communities, are disproportionately affected by lack of access to government-issued ID.^1,3,10–14^ Accordingly, government-issued IDs affect access to a broad range of resources linked with mental and physical well-being; unequal access to IDs is a health equity concern.

### Promoting health equity through local IDs

Given the importance of ID access, localities (county or city) across the United States have begun making local government-issued IDs available to local residents.^15,16^ Localities including New Haven, CT; San Francisco, CA; and New York City, NY have led the movement. To date, we are aware of 14 municipalities across 7 states and the District of Columbia who have begun issuing local IDs,^15–23^ with several other localities recently approving or considering similar policies.^24–27^ Local IDs hold promise for disrupting the health equity implications of restrictive ID policies. However, few local ID policies have been evaluated^5,17,28^ to ensure local IDs improve access to resources that are contingent upon having government-issued ID and unbiased treatment in acceptance of local IDs. Among the intended benefits of local ID policies is the reduction of racial/ethnic inequities linked with restrictive ID policies.

Local ID movements are a direct response to restrictive federal and state ID policies in an effort to enhance inclusion and equity in a domain whereby residents may organize to affect local decision-makers. The local ID movement shares some characteristics with “sanctuary city” discussions to challenge federal immigration enforcement initiatives directed by the Department of Justice and Department of Homeland Security. Although not all sanctuary cities have local IDs, similar to local ID movements, “sanctuary city” discussions and policy change initiatives have centered on local organizing and decision-making among local residents and authorities to affirm immigrant residents' identities.

### Washtenaw ID

The Washtenaw ID Task Force is a collaboration of community and governmental representatives. It was formed in 2012 after several community-based organizations recognized the challenge of lack of current government-issued ID that increasingly affected multiple communities. In Washtenaw County, these communities included immigrants, those who experience housing instability, lower income residents, transgender individuals, older adults, racial/ethnic minority residents, and persons who have been formerly incarcerated. The Washtenaw ID Task Force estimated that 42,000 county residents lacked a current state ID or driver's license.^29^

Based upon assessments of federal and state policy contexts, the Task Force recognized the need for a local government-issued ID. Washtenaw County includes multiple towns ranging from small to midsized, with residents often having community and employment-related ties in multiple towns across the county. Accordingly, the Task Force identified the need for an ID issued by the County. In 2015, Washtenaw County, Michigan implemented the Washtenaw ID, making it available to residents who establish their residence in the county and affirm their identity.^30^

The Washtenaw ID Task Force has overseen the planning, implementation, and improvement process for the Washtenaw ID. Throughout the organizing and planning process, Task Force members recognized the importance of evaluating the Washtenaw ID to inform assessments about the effectiveness and use of the Washtenaw ID. As several community members who were involved in planning discussions were also affiliated with a large research university in the region, we formed a community–academic partnership to evaluate the Washtenaw ID. This evaluation of the Washtenaw ID conducted by our research team found that several Washtenaw ID holders reported inconsistent acceptance of their Washtenaw IDs in retail, banking, and healthcare settings. Thus, it seemed as though the Washtenaw ID served its intended purpose at times, while at other times it did not.

For whom was the Washtenaw ID working? Community reports to the Washtenaw ID Task Force suggested that non-Latina/o white Washtenaw ID holders experienced more widespread acceptance of their Washtenaw IDs than Latina/o and non-Latina/o black Washtenaw ID users. Uneven acceptance of the Washtenaw ID would impede the effectiveness of this local policy intervention, with implications for health equity. An effective Washtenaw ID would work equally well for all residents, 71% of whom are non-Latina/o white, 12% of whom are non-Latina/o black, and 4% of whom are Latina/o.^31^ This is especially important because Washtenaw ID holders are disproportionately people of color. Although the Washtenaw ID Task Force has intentionally avoided collecting extensive data on the racial/ethnic identity of ID holders, among a sample of 251 applicants who applied for Washtenaw IDs in the first 6 weeks of the policy's implementation, 84% identified as Latina/o, whereas 8% identified as non-Latina/o white and 3% identified as non-Latina/o black.^18^

This study investigates whether the differential effectiveness of the Washtenaw ID extends from biased treatment of racial/ethnic minority groups, which the Washtenaw ID Task Force agreed needed to be examined in real-world contexts. Audit studies, or field experiments in which researchers train mystery shoppers to act as a consumer within a given market,^32,33^ have been useful for assessing racial bias in multiple sectors plagued by discrimination, including employment, housing, and healthcare.^34–38^ These studies are important for assessing racial bias in real-world contexts where service and experiences are contingent upon institutional practices and bureaucratic agents.^32,33,39^

We identified one study examining racial/ethnic differences in acceptance of a local government-issued ID (established in 2007) and an unofficial ID card among Latino and non-Latino white men who attempted to purchase goods with a check-in store settings in New Haven, Connecticut.^5^ Findings suggested that racial bias in carding disproportionately affected Latino men relative to non-Latino white men, and the local ID only partially mitigated these inequities.^5^

### Research questions and hypotheses

Following these methods,^5^ our community–academic partnership used an audit study design to examine whether Washtenaw County residents experience racial bias in (1) day-to-day encounters in which government ID is relevant and (2) acceptance of the Washtenaw ID. Specifically, we evaluated racial/ethnic variation in the experience of being asked for identification and acceptance of the Washtenaw ID while purchasing a standardized basket of goods in local stores. As the Washtenaw ID evaluation indicated that half (51%) of Washtenaw ID card holders were women, our audit study builds upon and extends the literature by examining the carding experiences of women in one of the first Midwestern communities to implement a local government-issued ID, nearly one decade after the implementation of the local ID in New Haven, Connecticut. Shoppers reflected the three largest racial/ethnic groups in Washtenaw County: Latina, non-Latina black, and non-Latina white. In addition, it was critical to evaluate how the ID was experienced in routine encounters, so rather than attempt to purchase goods with a check, this study involved an attempt to purchase standardized basket of goods with cash—an experience that aligns more closely with the communities of focus for this evaluation.

This study was guided by three research questions. First, we examined whether there are racial/ethnic differences in requesting ID. Building on findings from previous research^5^ and preliminary findings from the Washtenaw ID evaluation, we hypothesized that Latina and non-Latina black shoppers would be more likely than non-Latina white shoppers to be asked for identification.^5^ Second, we tested for racial/ethnic differences in acceptance of the Washtenaw ID among shoppers who are carded. We hypothesized that Latina and non-Latina black shoppers would be less likely than non-Latina white shoppers to have their Washtenaw IDs accepted by the store clerk or manager. Third, we examined whether there were racial/ethnic differences in store clerk or store manager nonpositive comments about the Washtenaw ID among those who are asked for an ID. We hypothesized that upon presenting the Washtenaw ID, Latina and non-Latina black shoppers would encounter more frequent nonpositive responses to the Washtenaw ID than non-Latina white shoppers.

## Methods

### Store sample

This audit study is part of a community–academic partnership between leaders of the Washtenaw ID Task Force and researchers affiliated with several universities. Since 2013, our partnership has sought to develop, implement, and evaluate the Washtenaw ID. Research partners identified a list of stores across the five largest towns in Washtenaw County that sold a pre-established set of goods (described hereunder). We categorized these stores into four types: national/large chain grocery stores (*n*=12), regional/local chain grocery stores (stores that may have multiple locations but are limited to southeast Michigan; *n*=8), convenience stores (*n*=11), and pharmacies (*n*=9). We also consulted with representatives of communities disproportionately affected by restrictive ID policies to identify additional stores for inclusion. We identified a total of 40 unique stores.

Each store was visited by at least one shopper from each of the three racial/ethnic groups, for a total of 130 visits. The University of Michigan and University of California, Irvine Institutional Review Boards classified this study as nonhuman subjects research and, therefore, was exempt.

### Standardized shopping basket

The standardized shopping basket included milk, cereal, paper towels, and alcohol (sparkling wine). We purposefully included alcohol because as an age-restricted item, shoppers would be asked to provide ID to verify their age. Basket contents were selected to reflect typical items purchased by individuals and households.

### Mystery shoppers and training protocol

Seven Washtenaw County residents (henceforth *shoppers*) were hired and trained to perform audit evaluations at stores throughout Washtenaw County in December 2016. Shoppers were women, ages 25–32 years, and diverse in their racial/ethnic identity (three identified as non-Latina black, two identified as Latina, and two identified as non-Latina white). Each shopper obtained her Washtenaw ID. To control for others' perceptions of socioeconomic status, shoppers were provided with nondescript black purses, used cash for all purchases, and were trained not to display their cell phone while shopping. Shoppers were provided with a nondescript digital watch and were asked to wear a sweater or light jacket and jeans when shopping. Each visited 9–22 stores.

**FIG. 1.**
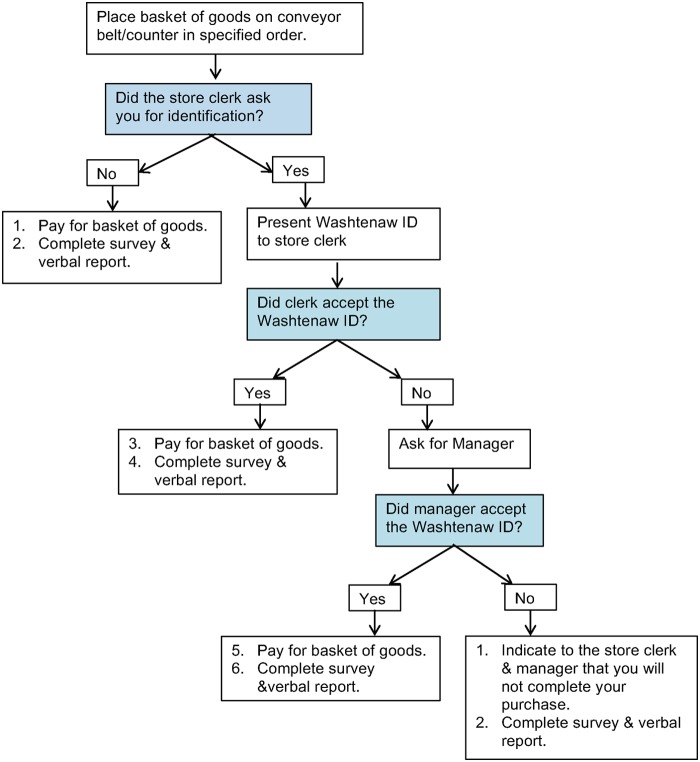
Mystery shopper protocol.

Shoppers completed a 4-h training regarding the shopping protocol (e.g., a script for interactions with store staff and completion of observation reports). The training included role-playing scenarios and interactions that might occur and recording observations in the standardized observational tools. Shoppers were trained to attempt to purchase the basket of goods at assigned stores. If asked to verify their age or show ID, shoppers would present their Washtenaw ID. If the Washtenaw ID was accepted, they would purchase the items. If the Washtenaw ID was rejected by the clerk and/or manager, shoppers would decline to purchase goods (see Fig. 1 for protocol for interaction with store staff).

### Variables

#### Measures

Shoppers completed standardized observational reports immediately after each shopping experience. This report included whether the clerk asked for their age or ID, the clerk's response to the Washtenaw ID, and whether the clerk accepted the Washtenaw ID. If clerks made a comment, in a closed-ended question, shoppers classified comments as: positive, negative, curious or inquiring, confused, neutral, asked where date of birth was marked on the ID, and/or said they do not accept the Washtenaw ID (check all that apply).

Shoppers received training regarding the classification of store clerks' comments. For example, an exclamation that store clerks were excited to see the Washtenaw ID be used or that Washtenaw County had a local ID was classified as positive comments; a comment that the Washtenaw ID did not look like a government-issued ID was characterized as a negative comment; questions about the implementation of the ID (e.g., “Who issues this ID?”) were categorized as curious or inquiring, questions such as “What is this?” were classified as neutral. Owing to the small sample size and the wide distribution of responses, we dichotomized these experiences (1=receipt of a comment that was negative, curious or inquiring, confused, neutral, asked about location of date of birth on the ID, or reported that the store does not accept the Washtenaw ID; 0=no comment or positive comment about the Washtenaw ID).

#### Dependent variables

We classified the experience of being asked for ID as being carded (1=carded, 0=not carded). When shoppers were carded, outcome variables were (1) whether the Washtenaw ID was rejected by the clerk or manager (1=rejected, 0=accepted) and (2) receipt of a nonpositive comment from the clerk or manager (1=nonpositive comment, 0=otherwise).

#### Independent variable

Shoppers' racial/ethnic identification, the key independent variable, was classified as Latina (1), non-Latina black (2), or non-Latina white (0).

#### Covariates

In sensitivity analyses, store type was included as a covariate (national grocery store (referent), regional/local grocery store, convenience store, and pharmacy).

### Analysis

We assessed means and frequencies to identify how best to include variables in the descriptive statistics and logistic regression analyses. To test the hypothesis of racial/ethnic variation in being carded, rejection of the Washtenaw ID, and receipt of comments about the Washtenaw ID, we used chi-square tests and logistic regression. In sensitivity analyses regarding carding experiences across store sites, in separate models, we regressed each of the outcome variables on the shopper's racial/ethnic identification and store type (available upon request).

## Results

Seven shoppers conducted a total of 130 shopping transactions, 30.8% of which took place at national grocery store chains, 22.3% at regional/local grocery stores, 20.8% at convenience stores, and 26.1% at pharmacies (Table 1). Shoppers were asked for an ID in 63.1% of transactions. Of these, the Washtenaw ID was accepted in 91.5% of cases.

**Table 1.** **Descriptive Statistics, Washtenaw ID Audit Study**

	Number of shopping transactions (*n*=130)	Percent of shopping transactions
Mystery shopper racial identification		
Latina	41	31.5
Black, non-Latina	49	37.7
White, non-Latina	40	30.8
Store type		
National grocery store	40	30.8
Regional/local grocery store	29	22.3
Convenience or party store	27	20.8
Pharmacy	34	26.1
Store town		
Ann Arbor	70	53.9
Other Washtenaw County town	60	46.1
Store clerk's perceived gender		
Woman	81	62.3
Man	49	37.7
Store clerk's perceived race		
Person of color	44	33.9
White, non-Latino	86	66.1
Carded		
Not asked for ID	48	36.9
Asked for ID	82	63.1
Of those asked for ID (*n*=82)		
Washtenaw ID accepted by store clerk or manager	75	91.5
Washtenaw ID rejected by store clerk or manager	7	8.5
Received nonpositive comment about Washtenaw ID	29	35.4
Did not receive comment about Washtenaw ID or received positive or neutral comment	53	64.6

ID, identification card.

**FIG. 2.**
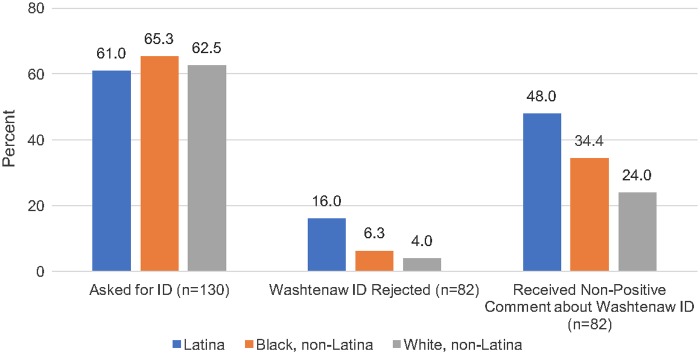
Percent of shoppers who were asked for ID, whose Washtenaw IDs were rejected, and who experienced a comment about their Washtenaw ID. ID, identification card.

In nearly 6 in 10 shopping experiences, non-Latina black (65.3%), non-Latina white (62.5%), and Latina (61.0%) shoppers were asked for ID (Fig. 2; *p*=0.91). We could not reject the null hypothesis of no racial/ethnic differences in experiences of being asked for ID for Latina (OR=0.94, *p*=0.89) or non-Latina black (OR=1.13, *p*=0.78) shoppers compared with non-Latina white shoppers (Table 2, Outcome 1).

**Table 2.** **Asked for ID, Rejected Washtenaw ID, and Received Comment About Washtenaw ID Regressed on Shoppers' Racial Identification**

	*B*	SE	OR	Lower 95% CI	Upper 95% CI	*p*
Outcome 1: Asked for ID (*n*=130)						
Intercept	0.51	0.33				
Racial identification (Ref: Non-Latina white)						
Latina	−0.06	0.46	0.94	0.38	2.30	0.89
Non-Latina black	0.12	0.44	1.13	0.47	2.69	0.78
Outcome 2: Washtenaw ID rejected (*n*=82)						
Intercept	−3.18	1.02				
Racial identification (Ref: Non-Latina white)						
Latina	1.52	1.16	4.57	0.47	44.17	0.19
Non-Latina black	0.47	1.25	1.60	0.14	18.72	0.71
Outcome 3: Comments about Washtenaw ID (*n*=82)						
Intercept	−1.15	0.47				
Racial identification (Ref: Non-Latina white)						
Latina	1.07	0.62	2.92	0.87	9.78	0.08
Non-Latina black	0.51	0.60	1.66	0.51	5.36	0.40

Referent groups include non-Latina white shoppers.

CI, confidence interval; OR, odds ratio; SE, standard error.

Among those who were asked for ID, a higher percentage of Latina (16.0%) shoppers had their Washtenaw ID rejected than black (6.3%) and white (4.0%) shoppers, although differences were not statistically significant (Fig. 2; *p*=0.27). Latina shoppers (OR=4.57, *p*=0.19) had over four times the odds of their Washtenaw IDs being rejected compared with non-Latina white shoppers, although the difference did not reach statistical significance because the subset of participants who were carded was smaller than the full sample (Table 2, Outcome 2). Non-Latina black shoppers (OR=1.60, *p*=0.71) were not significantly more likely to have their Washtenaw ID rejected compared with non-Latina white shoppers.

Finally, compared with non-Latina white (24.0%) and non-Latina black (34.4%) shoppers, a greater proportion of Latina shoppers (48.0%) received nonpositive comments about their Washtenaw IDs (*p*=0.21). Latina shoppers (OR=2.92, *p*=0.08) were 2.9 times more likely than non-Latina white shoppers to experience a nonpositive comment about their Washtenaw IDs, a pattern that was marginally significant (Table 2, Outcome 3). There was no statistically significant difference in receipt of a comment for non-Latina black (OR=1.66, *p*=0.40) versus non-Latina white shoppers. Sensitivity analyses accounting for store type did not change these findings.

## Discussion

The purpose of this audit study was to evaluate for whom the Washtenaw ID worked best and why. There are three key findings from this study. First, although results did not reach statistical significance, bivariate patterns suggest that Latina shoppers were more likely than white shoppers to have their Washtenaw IDs rejected, findings that mirrored previous results.^5^ Second, Latina shoppers were more likely than non-Latina white shoppers to experience nonpositive comments from a store clerk when presenting their Washtenaw IDs. These findings suggest that even when Latina shoppers' Washtenaw IDs are accepted in stores, they encounter more psychosocial barriers in the process of trying to use their Washtenaw IDs. Third, these findings suggest that the Washtenaw ID was accepted in 91% of observed retail interactions. In response to these findings, the Washtenaw ID Task Force is considering policy interventions and social campaigns to ensure that Latina/o Washtenaw ID holders can successfully use their Washtenaw ID without bias, in a context of highly racialized federal and state ID policies. We discuss these findings hereunder.

We hypothesized that the Washtenaw IDs of non-Latina white shoppers would be accepted at higher rates than those of racial/ethnic minority shoppers. Notably, there were differences in the experiences of Latina shoppers relative to both black and white shoppers. In *post hoc* thematic analyses of the nonpositive comments that Latina shoppers received, themes included an emphasis on citizenship status (e.g., “[We] only accept driver's licenses or state IDs, or if the Washtenaw ID has [your] passport number”), were racially coded (e.g., “So you're special. You have a special ID … [I] don't need any more IDs.”), and/or included acceptance of the ID while simultaneously undermining the validity of this government-issued ID (e.g., “Oh, what the hell!”). This is not to say that racism was only experienced by Latinas, but that the particular way in which racialization of Latinas/os is expressed was more aptly captured in these exchanges.

A growing literature points to the current and historic racialization of legal and citizenship status, with disparate impacts for Latina/o communities.^3,40–43^ Racialized immigrant policies, practices, and discourse are rooted in ideologies that characterize Latinas/os as perpetual foreigners, whose citizenship status and documentation are suspect.^3,41,44^ Thus, although differences in ID acceptance did not reach the level of statistical significance across racial/ethnic groups, Latina shoppers found their government documents more likely to be interrogated and negatively commented against. These discriminatory interactions can themselves change behavior, and can make ID-related interactions a location for anticipatory stress, or racism-related vigilance, which can take its own toll on health and well-being.^45^ That is, above and beyond differences in ID acceptance, negative commentary can inhibit individuals from repeated use of their Washtenaw IDs, therefore, creating a racially biased system and enhancing community distrust of an equity-driven community intervention. Furthermore, the act of questioning government paperwork is a symbolic interaction often linked to the threat of deportation.^1,3^ Therefore, negative commentary, although seen by outsiders as relatively harmless, can represent an interaction with a bureaucracy that has extremely negative consequences. This negative commentary—as well as the overt denial of the Washtenaw ID—also highlights the capacity of individual actors to disrupt community interventions. That is, even though the Washtenaw ID is a valid form of government ID that county representatives and the Sheriff's Department have endorsed, individual store clerks were still able to exercise their individual discretion and reject or question its use. Thus, successful community level interventions must also include limiting the individuals from discretionarily creating inequitable hierarchies on a smaller scale.

These findings suggest that the process of using IDs is more complex than simply being asked for ID. The extent to which shoppers were taken seriously when being carded suggests a pattern of racial/ethnic differences in opportunities to appeal rejection of the Washtenaw ID. Building upon these findings, the Washtenaw ID Project is planning to intervene on these racialized ID experiences by following up with stores that rejected the ID and/or where shoppers received biased comments. In addition, findings point to the need to actively and vigorously promote equity in access to and acceptance of the Washtenaw ID, with a focus on disrupting racialization processes that adversely affect racial/ethnic minority residents. Toward this end, the Washtenaw ID Project is considering the following: redesigning the Washtenaw ID, with a focus on racial equity in these considerations; reviewing the Washtenaw ID application process and requirements to ensure that the Washtenaw ID is accessible to all county residents; addressing application processes and costs that may impede access to the Washtenaw ID; and developing strategies to enable institutional stakeholders (e.g., store managers) to quickly learn about the validity of the Washtenaw ID when it is presented in their institutional domain.

Importantly, the Washtenaw ID Project is also considering social campaigns to enhance general awareness of the Washtenaw ID and to encourage allies with the privilege of having current U.S. government-issued photo ID to obtain and actively use their Washtenaw ID. In turn, allies' use of their Washtenaw ID would operate to disrupt the racialization of IDs by demonstrating that the Washtenaw ID is not only used by marginalized racial/ethnic minority groups.^46^ The Washtenaw ID Project is also considering strategies to encourage businesses, nonprofit organizations, and governmental organizations to accept the Washtenaw ID through campaigns that are centered on the county's identity as inclusive, innovative, and locally oriented.

### Limitations and strengths

Findings reported here should be considered within the context of several limitations. First, we examined racial bias in carding experiences and acceptance of the Washtenaw ID across one institutional setting: stores in which trained shoppers could purchase an age-restricted item. As stores have an established protocol for carding customers who attempt to purchase alcohol, our findings may mask the range of ways in which carding and ID acceptance surface in day-to-day experiences. Results may not be fully generalizable to other instances where a government-issued ID is needed to access resources such as housing or medication.

Second, this audit study was conducted over a 1-week period in December 2016. The evaluation was implemented during a particularly racialized sociopolitical moment, after a protracted period of racial politics throughout the 2016 presidential election and afterward. This period was characterized by a substantial rise in reported hate crimes across the United States and in Michigan.^47^ Accordingly we limited the audit study to a 1-week period to control for this racially volatile sociopolitical context. Findings should be understood as pertaining to this social and historical moment.

Third, funding to conduct this study was small, which limited the number of shoppers we could hire and train and potentially limited the study's statistical power. For example, several findings had large effect sizes (e.g., OR=4.57 for Latina shoppers having their ID rejected when compared with non-Latina white women) but failed to reach conventional levels of statistical significance (*p*<0.05). Therefore, it remains unclear whether a larger sample size would have resulted in statistically significant findings. Also, more research on this topic is needed to determine whether these findings can be replicated in other settings.

To implement an effective focused audit study, we limited sociodemographic variation in the group of shoppers, only examining experiences of women shoppers of a limited age range. We focused on women because the previous published audit study was conducted with men,^5^ 51% of Washtenaw ID holders who participated in the evaluation were women, and we wanted to observe an activity that is a part of day-to-day experiences for many Washtenaw ID holders. Future research should consider the experiences of a wider range of groups, and intersections such as race and gender, gender and age, and/or race and class.

Furthermore, we were only able to examine interactions in one specific setting (retail purchases), and not other health-relevant settings where ID might be requested (e.g., healthcare providers, banks, libraries, apartment applications, or food banks). We selected stores because there were enough retail settings in the single county where the policy was in effect to allow a large number of audit interactions, and it was a relatively low-barrier low-cost setting to implement a standardized interaction involving ID (as opposed to interactions at clinics, pharmacies or banks, which are harder to implement at the scale necessary for an audit study). Future research could examine racialized experiences presenting ID in these settings.

Notwithstanding the limitations, this study also has several strengths. The audit study design allowed investigators to observe day-to-day interactions without altering the environment through observation, providing the opportunity to evaluate this community-driven policy intervention in real-world contexts. Second, shoppers were trained in a standardized protocol that provided detail about the set of goods that they were to purchase, how to engage with store staff, and reporting observations. Third, we held constant several individual-level factors, including perceived age of shoppers, language use, perceived socioeconomic position, gender, and time period during a racialized sociopolitical context, allowing us to focus on racial bias in carding experiences and/or acceptance of the Washtenaw ID.

### Health equity implications

Audit studies are a promising evaluation strategy to ensure that equity-driven policy interventions improve social and health equity as intended. Findings suggest that local IDs may improve access to resources linked with having a government-issued ID *and* point to a need for continued efforts to mitigate the racialization of IDs. In the absence of reform to ensure access to state-issued IDs for all, continued attention is needed to evaluate whether initiatives to disrupt policies linked with health inequities are moving toward the goal of equity.

## Abbreviations Used

CI: confidence interval
ID: Government-issued photo identification card
OR: odds ratio
SE: standard error
